# Norwegian male U14 soccer players have superior running capacity compared to Icelandic players

**DOI:** 10.3389/fspor.2024.1407842

**Published:** 2024-07-01

**Authors:** Sigurður Benediktsson, Erlingur Johannsson, Cecilie Brekke Rygh, Hilde Gundersen

**Affiliations:** ^1^ Center of Sport and Health Sciences, School of Education, University of Iceland, Reykjavik, Iceland; ^2^ Department of Sport, Food and Natural Sciences, Western Norway University of Applied Sciences, Bergen, Norway; ^3^ Department of Health and Functioning, Western Norway University of Applied Sciences, Bergen, Norway; ^4^ Department of Radiology, Haukeland University Hospital, Bergen, Norway

**Keywords:** youth athletes, Iceland, Norway, physical performance, biological maturity, selected and deselected players

## Abstract

The organisation and development strategies of youth soccer differ between Norway and Iceland. Whether this affect physical capacity is unknown. Thus, the first aim of the present study is to compare physical capacity between players from Iceland and Norway. Secondary aim is to assess associations between biological maturity and physical capacity in the Icelandic players since an association previously has been shown among the Norwegians. There were 48 U14 players from Iceland included and 103 players from Norway. Bone age (BA), measured with left-wrist x-ray, was used as an indicator of biological maturity. To measure physical capacity, 40 metre (m) linear sprint, standing long jump (SLJ), countermovement jump (CMJ), the Yo-Yo intermittent recovery test (IR1-test) and a maximal oxygen uptake test (VO_2max_) were used. Training load was assessed by questionnaire. The results showed that the Norwegian players ran faster (5.90 ± 0.38 vs. 6.37 ± 0.44 s, *p* < .001), had better intermittent endurance capacity (1,235 ± 461 vs. 960 ± 423 m, *p* < .001) and higher VO_2max_, (60.3 ± 6.5 vs. 54.8 ± 5.3 ml·kg^−1^·min^−1^, *p* < .001) than the Icelandic players. The players from Norway reported a higher number of weekly organised soccer training hours than the Icelandic. We also found significant correlations between BA and performance on 40 m linear sprint (r = −.566, *p* < .001), SLJ (r = .380, *p* = .008) and CMJ (r = .354, *p* = .014) among the Icelandic players. Moreover, no correlations were found between BA and VO_2max_ or intermittent endurance capacity. In conclusion, the Norwegian players ran faster and had better VO_2max_ and intermittent endurance capacity than the Icelandic players. Biological maturity level was associated with speed and jumping performance in U14 soccer players in Iceland, but not with VO_2max_ or intermittent endurance capacity. Findings indicate that more research is needed to investigate the influence of different organisation and structure of youth soccer between the two countries on physical capacity.

## 1 Introduction

Soccer requires players to possess physical, technical, and tactical capabilities (1) due to its complexity. Despite the goal of developing successful soccer players at the elite senior level, the organisation and development strategies in youth soccer differ between countries (2–4). To succeed at the elite level, speed, intermittent endurance capacity and aerobic capacity at high level are required (5–9). The growth, biological maturity and physical capacity of male youth soccer players have received great attention in the literature (10–15). However, it remains unclear how various organizational and developmental strategies in youth soccer impact the development of physical capabilities among young athletes.

Both in Norway and Iceland, soccer is the most popular sport among children and youth. Players from both countries usually train with their local soccer club on teams with their schoolmates until the age of 13. After that age, the organisation differs between the countries. In Iceland, there is no strict differentiation between grassroots and elite youth soccer and the opportunity to join local soccer clubs is available for all youth soccer players regardless of their skills or training backgrounds, meaning that players at different levels are training together. The soccer clubs in Iceland do not have a traditional soccer academy for talent development, according to definitions from the Fédération Internationale de Football Association (FIFA) (16). Unlike in Iceland, most elite clubs in Norway recruit talented youth players from local soccer clubs to their academies at the age of 13–15 years, meaning that players at highest level train and play matches together as suggested by FIFA. Despite different organisational and developmental strategies for youth soccer, both Iceland and Norway have a strong passion for soccer and have achieved notable success on the international stage, making them interesting subjects for comparison. In 2023, both Iceland and Norway qualified for the male European U20 championship, showing that both countries have national junior teams at elevated levels.

Bone age (BA) is an important marker of biological maturation, in other words, puberty. In youth athletes, physical capacity is influenced by puberty, making talent identification and development challenging. Several studies have shown that biological maturity level is associated with physical capacities, indicating that more mature youth players have a physical advantage compared to their less mature peers (17–22). While the relationship between biological maturation and physical capacity has been established among Norwegian soccer players, it remains unexplored among Icelandic players. Thus, it is imperative to acknowledge the role of biological maturation when comparing physical capacities across different populations.

Comparing physical capacity among youth players from two countries with distinct soccer traditions is interesting, despite their small populations. Moreover, a comparison of physical capacities among youth soccer players from two different Nordic countries is lacking in the current literature. Based on current knowledge, the first aim of this study was to compare physical capacity between 14-year-old soccer players from Norway and Iceland. Considering the notably distinct structures and organization of youth soccer in Norway and Iceland, our hypothesis posits that the physical capacities of 14-year-old soccer players from these two Nordic countries would differ. Secondly, we aimed to assess the association between biological maturity and physical capacity in 14-year-old Icelandic soccer players. Our second hypothesis is to confirm the association between biological maturity and physical capacity in 14-year-old Icelandic soccer players as have been shown in the Norwegian players.

## 2 Materials and methods

The present study was a collaboration between researchers at the University of Iceland, Reykjavik, Iceland, and Western Norway University of Applied Sciences, Bergen, Norway. The data included in this study for the U14 players in Norway was collected in June 2018 as part of a longitudinal research project examining factors related to talent development in youth soccer. The results from the Norwegian sample have previously been published for other purposes (19, 20, 23). Data from Iceland was collected from February to May 2021. All tests were conducted indoors with experienced testing personnel. The same test protocol was used in both countries.

### 2.1 Participants

In total, 151 U14 male soccer players were included in this study. The Norwegian players (*n* = 103) were recruited from seven local U14 soccer teams. Among the Norwegian players, 80 were selected to local academy teams before the U14 season, whereas 23 played on lower-level teams, defining them as deselected players. The Icelandic players (*n* = 48) were recruited from two local U14 soccer clubs. Since there are no academies in Iceland, both teams included players at all levels.

Both the National Bioethics Committee of Iceland (VSN-19-206) and the Regional Committee for Medical and Health Research Ethics in Norway (2017/1731) approved the study, and the study was conducted in accordance with the Helsinki Declaration. Because players were under the legal age of consent, both the players and their parents gave written informed consent for participation. Results were treated anonymously for all participants.

### 2.2 Anthropometric data

Anthropometric measurements included height and weight. Height was measured with a stadiometer (Seca 206 and Seca 217, Hamburg, Germany). Measurements were performed barefoot using standard procedures. In Norway, body weight was assessed with an eight-polar bioimpedance method using multifrequency current (InBody™ 720, Biospace Co.). In Iceland, body weight was assessed using a digital weight scale (FG-150KAL, A&D Company, limited).

### 2.3 Physical tests

In Norway, 40 m linear sprint, standing long jump (SLJ) and the Yo-Yo intermittent recovery test (IR1-test) were performed on the first test day, whereas countermovement jump (CMJ), and maximal oxygen uptake (VO_2max_) were assessed on the second test day. The same order of tests was completed in Iceland, except for an additional test day for the IR1-test for practical reasons. The players were wearing t-shirts, along with shorts and indoor sports shoes during the tests.

### 2.4 40-m linear sprint

A 40 m linear sprint test was conducted to measure the player's speed. All players conducted a standardized 30-min warm-up protocol before the test, led by a physical trainer. The warm-up consisted of 10-min low-intensity running, followed by 5 min of dynamic stretching. In the final part of the warm-up, players performed 4 × 40–50 m linear runs with increasing intensity and speed, followed by two maximal linear accelerations of 20 m.

After the warm-up, all players performed three maximal sprints of 40 m separated by 2–3 min of rest. The players started from a standing position with split legs, with the toes of the front foot placed 60 cm behind the first photogates. The fastest sprint of three attempts was included in the analysis (24).

A wall-mounted photogate system (IC Control TrackTimer) was used in Norway, whereas a portable photogate system (Witty, Microgate, Italy) was used in Iceland. The height of the first photogate was 50 cm above the running surface, while the photogates at 10, 20, 30, and 40 m were positioned 120 cm above the running surface (24).

### 2.5 Jump performance

#### 2.5.1 Standing long jump

Standing long jump (SLJ) was performed to measure the player's explosive strength in the horizontal plane. Players started with both feet placed behind a line marked on the ground and jumped as long as possible in a forward direction. Players were not allowed to move their feet before jumping. Players had magnesium under the soles of their shoes to identify the landing mark. The horizontal distance from the start line to the mark made by the heel closest to the start line was measured and used to determine the jumping length. The jump was not approved if the player touched the ground with one of their hands. The best of three attempts was used in the analyses.

#### 2.5.2 Countermovement jump

Countermovement jump (CMJ) was performed to measure the players explosive strength in the vertical plane. Players started in a standing position with their arms on their hips, and with hips and legs extended. Players were instructed to jump as high as possible, after an initial knee flexion. They were also instructed to have straight legs in the air. Instructions on jump technique and trail jumps were given to ensure that players were familiar with the jumping technique. The best of three attempts was used in the analyses. The CMJ test was performed with the Kistler 9286B force plate (Kistler Instruments AG) in Norway and an optical measurement system with a sampling frequency of 1,000 Hz (Optojump RX 10, Microgate, Italy) in Iceland. Maximum jump height in centimetres (cm) was calculated from take-off velocity in Norway and from flight time in Iceland.

### 2.6 Aerobic capacity and intermittent endurance capacity

#### 2.6.1 Maximal oxygen uptake

To determine VO_2max_, players ran on the constant inclination of 5.3% on a motorized treadmill (Woodway PPS55, USA). The inclination of the treadmill was used to limit the effect of the running technique on test performance. The test protocol started with a speed between 7 and 10 km h^−1^, with individual starting speeds decided for each player after the warm-up. After the start of the test, the speed was increased by 1 km h^−1^ every minute to voluntary exhaustion. The VO_2max_ was determined using the highest average of two consecutive 30-s measurements (ml·kg^−1^·min^−1^). Players' perceived exertion on the Borg scale was registered within 30 s after the test was finished.

Before the test, each player performed a 10-min warm-up on a treadmill, with a gradual increase in speed. Heart rate (HR) was measured with an HR monitor (Polar V800, Polar Electro OY). The volume of oxygen (VO_2_) was measured using a computerized metabolic system with a mixing chamber (Oxycon Pro, Erich Jaeger GmbH). The flowmeter was calibrated with a 3-L volume syringe (Hans Rudolph, Inc.) before each test. The VO_2_ and carbon dioxide (VCO_2_) using high-precision gases were also calibrated (16.00 ± 0.04% O_2_ and 5.00 ± 0.1% CO_2_, Riessner-Gase GmbH & co, Lichtenfels, Germany).

#### 2.6.2 The Yo-Yo intermittent recovery test

The Yo-Yo intermittent recovery test (IR1-test) was used to assess the players' intermittent endurance capacity. Standardized procedures were conducted, with a standard starting speed for all players (25). The total distance covered in metres was used for statistical analysis. In Norway, the test was performed on an indoor wooden sports floor, while in Iceland, players ran the test in an indoor soccer arena with artificial grass.

### 2.7 Questionnaire data

All players answered questions regarding the number of sessions and hours of weekly organised soccer training and the age at which they started to play organised soccer.

### 2.8 Bone age

Players underwent an x-ray of their left wrist to estimate skeletal and biological maturity based on their bone age (BA). The x-ray images were obtained using Siemens Yasi Max with the integrated imaging system FLUORPSPO Compacts (software version VE10; Siemens Healthineers). The field of view covered the hand, as well as 3 cm of the lower distal arm to include the epiphyseal plates in the radius and ulna. The parameters were as follows: tube-detector distance 1 m, x-ray energy 50-kilo volt (kV), and 1–1.5 milliampere seconds (mAs), with no processing or filtering of the images.

BoneXpert standalone version 2.5 (Visiana) was used to analyse the radiographs. The system automatically performs 8–13 independent BA measurements from 8 to 13 different bones in the left hand. The automated determination of BA rules out inter- and intra-observer variation. This BA determination was based on the Greulich Pyle methodology (26).

### 2.9 Statistical analyses

Descriptive data is shown as mean ± standard deviation (SD) and minimum (min) and maximum (max) values. The normality of data was assessed with the Shapiro-Wilkins test. One-way ANOVA analysis was performed to evaluate differences between the Icelandic and the Norwegian players regarding chronological age (CA), BA, height, weight, and number of weekly organised training sessions and hours. Because previous studies have shown a relationship between BA and physical capacity, and since we found a significant difference in BA between the Norwegian and Icelandic players one-way analysis of covariance (ANCOVA) was performed to compare the physical capacities between the Icelandic and Norwegian players, with BA as a covariate. The least significant difference (LSD) *post hoc* analyses were performed to compare the Icelandic players with the selected and deselected Norwegian players. Data presented in the Tables are unadjusted. The relationship between variables was assessed using Pearson's correlation coefficient *r*. An r -value between 0.01 and 0.29 was defined as a small correlation, between 0.30 and 0.49 as a medium correlation, and from 0.05 to 1.0 as a large correlation (27). The Statistical Products of Service Solution package (SPSS, version 29) was used for all statistical analyses, and *p*-values of ≤0.05 were considered statistically significant.

## 3 Results

### 3.1 Descriptive data

Mean CA of the players (*n* = 151) was 14.0 ± 0.3 years, whereas mean BA was 13.9 ± 1.0 years. When the Norwegian group was divided into the selected and deselected groups, no significant differences were observed between the groups regarding BA, date not shown. Mean body height and weight were 166.8 ± 8.6 cm and 53.8 ± 8.9 kg, respectively. For an overview of the characteristics of the players from Iceland and Norway separately, see Table 1.

**Table 1:** An overview of characteristics of the Icelandic and Norwegian U14 soccer players. Data are represented as mean ± SD.

	Norwegian players (*n* = 103)	Icelandic players (*n* = 48)	*p*-value
Chronological age (years)	14.1 ± 0.3	13.9 ± 0.3	<.001
Bone age (years)	14.0 ± 1.1	13.6 ± 0.7	.016
Height (cm)	167.3 ± 8.4	165.6 ± 8.8	.253
Weight (kg)	54.0 ± 7.2*	52.6 ± 7.2	.370

* *n* = 98.

The Icelandic and Norwegian players reported that they started with organised soccer training at an age of 5.5 ± 2.1 years and 5.8 ± 1.0 years, respectively. There was no significant difference between players from the two countries regarding when they started to play soccer.

### 3.2 Physical capacities between players from Iceland and Norway

Significant differences in speed and endurance capacity were seen between the Norwegian and Icelandic players (Table 2). Overall, the analysis showed that players from Norway ran faster, had higher VO_2max_ and ran longer in the IR1 recovery test. There was no significant difference between players from the two countries in SLJ and CMJ. ANCOVA analysis showed that the differences between players from Iceland and Norway were consistent also after adjusting for BA.

**Table 2:** An overview of the physical capacities and the weekly organised soccer training volume among the Icelandic and Norwegian U14 players. Data are represented as mean ± SD (min–max).

	Norwegian players	Icelandic players	*p*-value
Physical tests			
40 m linear sprint (sec)	5.90 ± 0.38 (5.11–7.41) (*n* = 103)	6.37 ± 0.44 (5.61–7.33) (*n* = 48)	<.001
SLJ (m)	1.88 ± 23 (1.25–2.58) (*n* = 95)	1.85 ± 22 (1.39–2.25) (*n* = 48)	.849
CMJ (cm)	29.1 ± 5.1 (14.9–39.4) (*n* = 97)	28.9 ± 4. (21.1–42.1) (*n* = 48)	.565
VO_2max_ (ml·kg^−1^·min^−1^)	60.3 ± 6.5 (38.5–75.6) (*n* = 95)	54.8 ± 5.3 (45.1–66.9) (*n* = 48)	<.001
IR1-test (m)	1,235 ± 461 (200–2,280) (*n* = 87)	960 ± 423 (280–1,920) (*n* = 48)	<.002
Organised weekly soccer training			
Sessions (*n*)	4.33 ± 1.65 (*n* = 96)	4.63 ± 1.20 (*n* = 48)	.277
Hours (*n*)	6.80 ± 3.41 (*n* = 97)	5.78 ± 1.39 (*n* = 48)	.049

SLJ, standing long jump; CMJ, countermovement jump; VO_2max_, maximal oxygen consumption; IR1-test, the Yo-Yo intermittent recovery test.

When splitting the Norwegian players into selected players and deselected players, the selected Norwegian players were significantly faster and had higher VO_2max_ than both the deselected Norwegian players and the Icelandic players, with no significant difference between the deselected Norwegian players and the Icelandic players. In the 40-meter test, IR1-test and V*˙*O_2max_ test, the selected Norwegian players performed significantly better than the deselected Norwegian players and the Icelandic players. In addition, the Icelandic players performed significantly better than the deselected Norwegian players in IR1-test and V*˙*O_2max_ test. In CMJ, both the selected Norwegian players and the Icelandic players were superior to the deselected Norwegian players, and there were no significant differences between the players in SLJ (Table 3).

**Table 3:** An overview of the physical capacities and the weekly organised soccer training among the Norwegian and Icelandic players when splitting the Norwegian players in a selected and deselected group. Data are represented as mean ± SD. (min–max).

	Norwegian selected players (1)	Norwegian deselected players (2)	Icelandic players (3)	*p*-value	*Post hoc* (LSD)
Physical tests					
40 m (sec)	5.82 ± 0.33 (5.11–6.61) (*n* = 80)	6.17 ± 0.42 (5.50–7.41) (*n* = 23)	6.37 ± 0.44 (5.61–7.73) (*n* = 48)	<.001	1 > 2,3
SLJ (cm)	191 ± 22 (140–258) (*n* = 72)	179 ± 25 (125–227) (*n* = 23)	185 ± 22 (139–225) (*n* = 48)	.071	
CMJ (cm)	29.9 ± 4.8 (20.3–39.4) (*n* = 77)	25.9 ± 5.3 (14.9–36.1) (*n* = 20)	28.9 ± 4.3 (21.1–42.1) (*n* = 48)	.002	1 > 2 3 > 2
VO_2max_ (ml·kg^−1^·min^−1^)	61.6 ± 5.7 (48.3–75.6) (*n* = 75)	55.6 ± 7.2 (38.5–67.9) (*n* = 20)	54.8 ± 5.3 (45.1–66.9) (*n* = 48)	<.001	1 > 2,3
IR1 (m)	1,329 ± 418 (520–2,280) (*n* = 75)	653 ± 235 (200–1,080) (*n* = 12)	960 ± 423 (280–1,920) (*n* = 48)	<.001	1 > 2,3 3 > 2
Organised weekly soccer training					
Sessions (*n*)	4.85 ± 1.44 (*n* = 75)	2.48 ± 0.75 (*n* = 21)	4.63 ± 1.20 (*n* = 48)	<.001	1 > 2 3 > 2
Hours (*n*)	7.83 ± 3.17 (*n* = 75)	3.27 ± 0.92 (*n* = 22)	5.78 ± 1.39 (*n* = 48)	<.001	1 > 2,3 3 > 2

SLJ, standing long jump; CMJ: countermovement jump; VO_2max_, maximal oxygen consumption; IR1-test, the Yo-Yo intermittent recovery test. >: indicate significant better performance.

### 3.3 Organised soccer training volume between players from Iceland and Norway

In general, there was no significant difference in the number of organised weekly training sessions between players from Iceland and Norway (Table 2). However, when splitting the Norwegian players into selected and deselected players, both the selected Norwegian players and the Icelandic players reported significantly higher number of weekly organised soccer training sessions compared to the deselected Norwegian players (Table 3). Moreover, the Norwegian players reported a significantly higher number of organised weekly training hours than the Icelandic players (Table 2). Analyses also showed that the selected Norwegian players had a significantly higher number of organised weekly training hours than both the Icelandic players and the deselected Norwegian players. However, the Icelandic players reported a significantly higher number of organised weekly training hours compared to the deselected Norwegian players (Table 3).

### 3.4 Relationships between BA and physical capacities among Icelandic players

Among the Icelandic players, there was a strong correlation between BA and performance on the 40 m linear sprint test (r = −0.566, *p* < 0.001), and medium correlations between BA and jumping performance (SLJ: r = 0.380, *p* = 0.008, CMJ: r = 0.354, *p* = 0.014). On the other hand, no significant correlations were observed between BA and performance on the IR1-test (r = 0.206, *p* = 0.161) nor the V*˙*O_2max_ test (r = 0.125, *p* = 0.369).

## 4 Discussion

In the present study, associations between BA and physical capacities in Icelandic U14 soccer players were investigated, and the physical capacity level and the volume of weekly organised soccer training between Icelandic and Norwegian youth soccer players were compared. The results showed that the Norwegian players were faster, had higher intermittent endurance capacity and higher V*˙*O_2max_ than the Icelandic players. Moreover, the Norwegian players reported a significantly higher number of weekly training hours than the Icelandic players, on average. In addition, BA of the Icelandic players was associated with speed and maximal jump performance, but not with intermittent endurance capacity or V*˙*O_2max_.

When comparing the U14 players from Iceland and Norway, our findings showed, after adjusting for biological maturity level, that the Norwegian players ran significantly faster than the Icelandic players on the 40 m linear sprint test. Overall, the Norwegian players also had significantly higher VO_2max_ and performed significantly better on the intermittent endurance capacity test compared to the Icelandic players. However, when splitting the Norwegian players into selected and deselected players, only the selected Norwegian players were superior to the Icelandic players when it comes to speed, intermittent endurance capacity and V*˙*O_2max_.

Differences in running capacity between the Norwegian selected players and Icelandic players may be caused by different reasons. One possible explanation refers to the different organisation of youth soccer in the two countries. Norwegian soccer academies may provide a more conducive training environment with high-level coaches, skilled teammates, and better training facilities, which can result in increased motivation and improved physical capacities (28). The organisation of youth soccer and talent identification in Norway correspond to general recommendations by FIFA, such as investment in the professionalisation of academies, when it comes to the selection of players (16).

Faster running speed and better physical capacity among the selected Norwegian players may be related to the selection of players *per se*, i.e., players with outstanding physical capacity are more likely to be selected to academies (19, 29), although previous studies have shown inconsistent and conflicting findings regarding physical fitness and selection to academies. The higher number of organised weekly training hours seen among the selected Norwegian players could also be an explanation. As mentioned before, the selected Norwegian players may also have higher training intensity due to higher competition among players and superior training motivation. Further research and assessments are needed. On the other hand, there was no significant difference between the deselected Norwegian players and the Icelandic players in 40 m linear sprint time and maximal oxygen consumption, despite the significantly higher number of organised training hours among the Icelandic players.

Our results showed a relationship between BA and running speed and jumping performance among the Icelandic players. This is in accordance with previous findings among Norwegian players at the same age (19, 20) and confirms previous findings regarding the effect of biological maturation on speed and explosive leg strength (17, 18, 21, 22). Our findings indicate that biological maturity level affects physical capacity among Icelandic youth soccer players, as has been demonstrated in other countries. Furthermore, there was no relationship between BA and endurance capacity among the Icelandic players. This is also in line with previous findings among Norwegian U14 soccer players (19, 20), and indicates that endurance capacity is not influenced by biological maturity status in highly trained youth soccer players. However, a recent investigation suggests that the positive change in VO_2max_ was paralleled by the increase in fat-free mass. Therefore, an increase in endurance capacity may be more related to body composition development, growth and maturation rather than different intensity or volume of the training (30). This finding indicates that physical training for youth soccer players needs to be investigated further in more detail in the future.

The present study has some limitations that are worth mentioning. The Icelandic measurements were conducted during the end of the COVID-19 pandemic, which presented unexpected obstacles, such as training bans, quarantine and additional limitations when measuring the youth soccer players. During the late COVID-19 period very limited training restrictions occur amount youth soccer players in Iceland. However, this may have affected the players' results in the various measurements and might skew results. It is also worth mentioning that different equipment was used for the 40 m sprint test and the CMJ test in the two countries. In the CMJ test, maximum jump height was calculated based on take-off velocity in Norway, while in Iceland it was derived from flight time. To ensure reliable results, only jumps with correct technique were considered valid (i.e., players were instructed to maintain straight legs in the air for accurate flight time). The portable photogates employed for the 40 m sprint test in Iceland were set up by experienced personnel, and there is no reason to suspect that the variance in running speed was influenced using a portable system in Iceland vs. the wall-mounted system in Norway. A strength of the study was that experienced personnel administered all tests. However, a limitation was the use of different photogate systems for the 40 m sprint test. Moreover, the IR1-test was done on different surfaces in the two countries. In Norway, the test IR1 test was conducted indoors on a wooden sports floor, while the test was conducted indoors on artificial grass in Iceland. It cannot be ruled out that different running surfaces may have had an impact on the test results. However, it is unlikely that the COVID-19 pandemic, the use of different equipment and different running surface alone can explain the difference between the Norwegian and the Icelandic players.

## 5 Perspective

In this study we compare physical capacity between U14 soccer players from Iceland and Norway. The main results showed that the Norwegian players had exhibited superior endurances capacity and ran faster than the Icelandic players. Furthermore, the Norwegian players reported a higher number of weekly organised soccer training hours than the Icelandic. These results are interesting, because the Fédération Internationale de Football Association (FIFA) highly recommended the soccer clubs to utilise the professional soccer academic during the process of the identification and talent development of the youth soccer players. In Norway, many of the promising young soccer players are selected to the academies at an age of 13, on the other hand, the traditional academies are not established or utilised in Iceland. However, the success of the Iceland men senior soccer team has been remarkable during the last decades, which indicate great potency of the Icelandic system. Furthermore, both Iceland and Norway were qualified for the male European U20 championship in 2023, showing that both nations can reach high international junior levels. Further research is needed to investigate the relationship between development of physical capacity, training load and different organisational structures of both youth and senior soccer players in Scandinavian.

## Data Availability

The raw data supporting the conclusions of this article will be made available by the authors, without undue reservation.
